# Complete chloroplast genome sequence and phylogenetic analysis of *Populus deltoides* Caihong

**DOI:** 10.1080/23802359.2020.1869612

**Published:** 2021-02-08

**Authors:** Weibing Zhuang, Xiaochun Shu, Hui Zhang, Tao Wang, Fengjiao Zhang, Ning Wang, Zhong Wang

**Affiliations:** a Jiangsu Key Laboratory for the Research and Utilization of Plant Resources, Institute of Botany, Jiangsu Province and Chinese Academy of Sciences (Nanjing Botanical Garden Mem. Sun Yat-Sen), Nanjing, PR China; b Forestry Scientific and Technical Extension Center, Lianshui, PR China

**Keywords:** Caihong poplar, *Populus deltoides*, chloroplast genome, whole-genome sequencing, phylogenetic analysis

## Abstract

Colored-leaf plants are increasingly popular, which has higher ecological, economic and social benefits. Caihong poplar, one of colored-leaf plants from *Populus deltoides*, has been widely used in courtyard embellishment, road greening, garden set King and so on. In this study, the complete chloroplast genome of Caihong poplar was evaluated, and the total chloroplast genome size of which is 156,957 bp in length with 36.69% GC content, including large single-copy region (LSC) of 85,096 bp, a pair of inverted repeat regions (IRs) of 27,649 bp each, and a small single-copy region (SSC) of 16,563 bp. There were 22 *tRNA* genes, 83 protein-coding genes, and four *rRNA* genes. The phylogenetic analysis with 22 species indicated that Caihong poplar was closely clustered with *Populus deltoides* Zhonglin 2025. In conclusion, the complete chloroplast genomes of Caihong poplar in this study provided valuable genomic resources for further phylogeny and species identification in the *Populus* family.

Colored-leaf plants are increasingly popular in modern society, which has higher ecological, economic, and social benefits. Caihong poplar, one of colored-leaf plants from *Populus deltoides*, has been widely used in courtyard embellishment, road greening, garden set King and so on. The chloroplast genome is a good method to study the mechanisms of plant biology, diversity, evolution and climatic adaptation, and genetic engineering according to the following reasons (Duan et al. 2020). First, the chloroplast genome is highly conserved in the organization, gene order and content. Second, the chloroplast contains its own genome, and the genes of which are single-copy, avoiding the interference of side-line homologous genes. Thirdly, it is cheaper and convenient to acquire the complete cp genome sequences now than ever. Although many chloroplast genomes in *Populus* have been sequenced, while the complete chloroplast genome sequence is not available for Caihong poplar. Recently, the complete chloroplast genome sequence of *Populus deltoides* Zhonglin 2025 has been characterized (Zhuang et al. 2020). Although Zhonglin 2025 and Caihong poplar are belonging to the *Populus deltoides*, it is still worth evaluating the complete chloroplast genome sequence of Caihong poplar to explore its physiological, molecular, and phylogenetical mechanism.

*Populus deltoides* Caihong was collected in the Nanjing Botanical Garden, Mem. Sun Yat-sen (E118_83, N32_06), Nanjing, China, and the voucher specimen were deposited there under accession number of SAMN 16401422. The genomic DNA from fresh leaves of Caihong was obtained by the DNeasy plant mini kit (Qiagen, Hilden, Germany). A paired-end library with an insert-size of 350-bp was constructed and sequenced on the Illumina NovaSeq system (Illumina, San Diego, CA). A total of 8143.6 Mb raw data were generated, and 7924.7 Mb clean data were used for the chloroplast genome reconstruction. *De novo* genome assembly and annotation were conducted by NOVOPlasty (Dierckxsens et al. 2017) and GeSeq (Tillich et al. 2017), respectively, and the chloroplast sequence of *P. trichocarpa* (NC_009143.1) was used as a reference (Tuskan et al. 2006). The raw sequencing reads used in this study were deposited in a public repository SRA, and the accession number is PRJNA668228. The annotated chloroplast genome was deposited in GenBank (accession number: MW165890).

The complete chloroplast genome of Caihong poplar was evaluated, and the total chloroplast genome size of which is 156,957 bp in length with 36.69% GC content, including large single-copy region (LSC) of 85,096 bp, a pair of inverted repeat regions (IRs) of 27,649 bp each, and a small single-copy region (SSC) of 16,563 bp. There were 22 *tRNA* genes, 83 protein-coding genes and four *rRNA* genes. To reveal the phylogenetic relationship of Caihong poplar with other members in *Populus*, a phylogenetic analysis was performed based on 18 complete chloroplast genomes from *Populus* and three taxa from *Salix*, and *Vitis vinifera* was served as outgroup. The maximum likelihood (ML) bootstrap analysis with 1000 replicates was performed using RaxML version 8.2.12 (Stamatakis 2014), and the sequences were aligned by MAFFT version 7.309 (Katoh and Standley 2013). The phylogenetic tree showed that *P. deltoides* Caihong was closely related to *P. deltoides* Zhonglin 2025 (Figure 1). The newly characterized *P. deltoides* Caihong complete chloroplast genome will provide essential data for further study on the phylogeny and evolution of the genus *Populus*, and provide useful resources for better understanding the physiology and evolution of the genus *Populus*.

**Figure 1.**
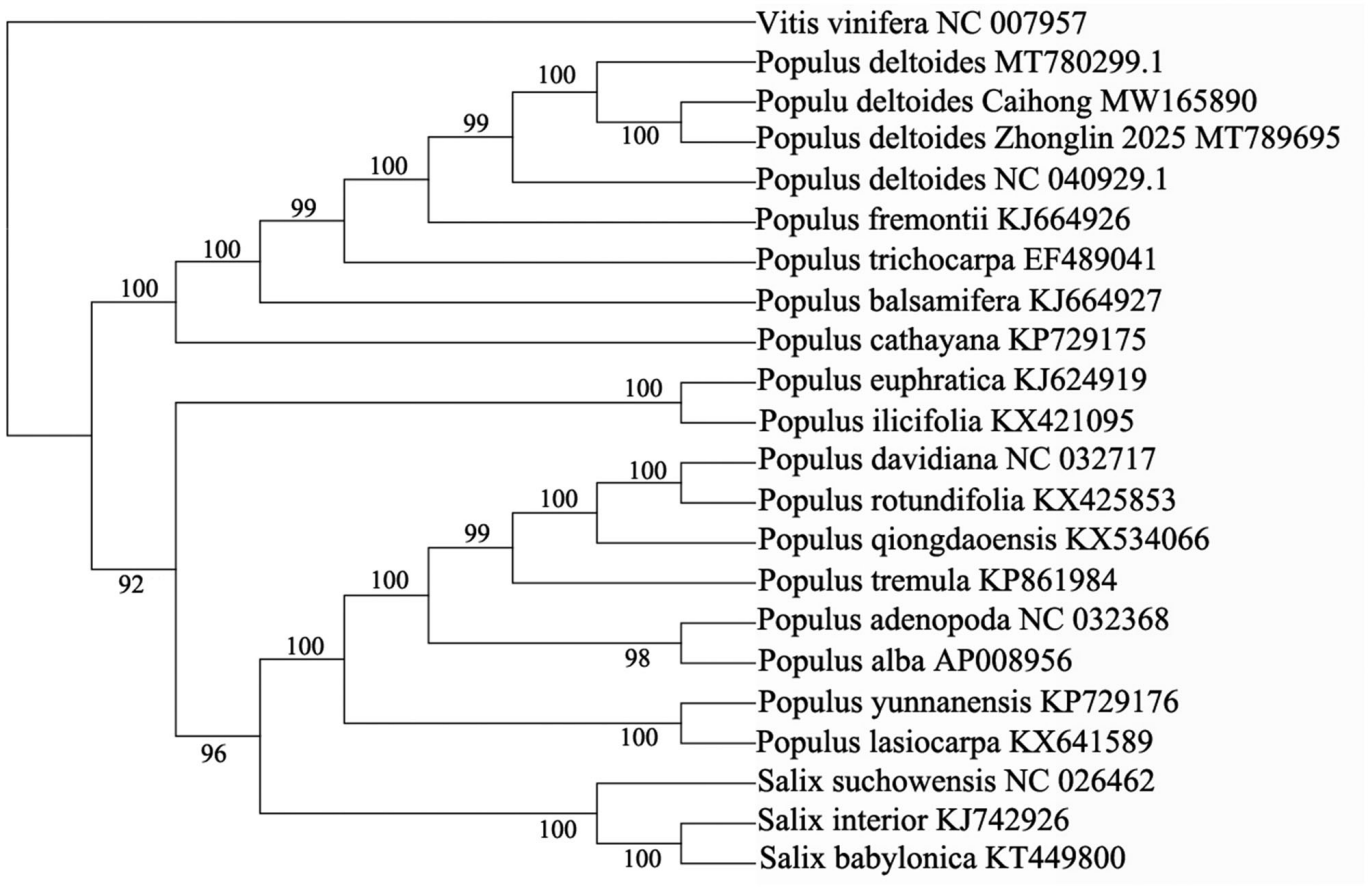
Phylogenetic tree was built with maximum likelihood (ML) bootstrap analysis based on 22 chloroplast genome sequences and three taxa from Salix, and *Vitis vinifera* were served as outgroups. Bootstrap support values (%) are indicated in each node.

## Data Availability

The genome sequence data that support the findings of this study are openly available in GenBank of NCBI at (https://www.ncbi.nlm.nih.gov/) under the accession no. MW165890. The associated BioProject, SRA, and Bio-Sample numbers are PRJNA668228, SRR12822488, and SAMN16401422, respectively.
